# The Molecular Epidemiology of Resistance to Antibiotics among *Klebsiella pneumoniae* Isolates in Azerbaijan, Iran

**DOI:** 10.1155/2021/9195184

**Published:** 2021-07-12

**Authors:** Mehdi Kashefieh, Hassan Hosainzadegan, Shabnam Baghbanijavid, Reza Ghotaslou

**Affiliations:** ^1^Student Research Committee, Tabriz University of Medical Sciences, Tabriz, Iran; ^2^Infectious and Tropical Diseases Research Center, Tabriz University of Medical Sciences, Tabriz, Iran; ^3^Department of Bacteriology and Virology, Faculty of Medicine, Tabriz University of Medical Sciences, Tabriz, Iran; ^4^Department of Microbiology, Faculty of Medicine, Maragheh University of Medical Sciences, Maragheh, Iran; ^5^Central Laboratory of the Province, Tabriz University of Medical Sciences, Tabriz, Iran

## Abstract

**Introduction:**

*Klebsiella pneumoniae* (*K. pneumoniae*) is one of the leading causes of hospital-acquired and community-acquired infections in the world. This study was conducted to investigate the molecular epidemiology of drug resistance in clinical isolates of *K. pneumoniae* in Azerbaijan, Iran.

**Materials and Methods:**

A total of 100 nonduplicated isolates were obtained from the different wards of Azerbaijan state hospitals, Iran, from 2019 to 2020. Antibiotic susceptibility testing was done. The DNA was extracted, and the PCR for evaluation of the resistance genes was carried out.

**Results:**

The highest antibiotic resistance was shown to ampicillin (96%), and the highest susceptibility was shown to tigecycline (9%), and 85% of isolates were multidrug resistant. The most frequent ESBL gene in the tested isolates was *bla*_SHV-1_ in 58%, followed by *bla*_CTXM-15_ (55%) and *bla*_SHV-11__(_42%). The *qepA*, *oqxB*, and *oqxA* genes were found to be 95%, 87.5%, and 70%, respectively. We detected *tetB* in 42%, *tetA in* 32%, *tetD* in 21%, and *tetC* in 16%. Seventy isolates were resistant to co-trimoxazole, and the rate of resistance genes was *sul*1 in 71%, followed by *sul*2 (43%), *dfr (*29%), and *sul*3 (7%). The most common aminoglycoside resistance genes were *ant*3*Ia, aac*6*Ib, aph*3*Ib*, and *APHs* in 44%, 32%, 32%, and 31.4%, respectively. The most frequent resistance gene to fosfomycin was *fosA* (40%) and *fosX* (40%) followed by *fosC* (20%).

**Conclusion:**

The results of this study indicate the high frequency of drug resistance among *K. pneumoniae* isolated from hospitals of Azerbaijan state. The present study shows the presence of high levels of drug-resistant genes in various antibiotics, which are usually used in the treatment of infections due to *K. pneumoniae*.

## 1. Introduction

*Klebsiella pneumoniae (K. pneumoniae)* is one of the prominent causes of hospital-acquired and community-acquired infections worldwide [1–4]. This bacterium is the second most common cause of urinary tract infections [1]. In comparison to classic nonvirulent isolates, hypervirulent *K. pneumoniae* strains affect healthy individuals with dangerous and invasive infections, while classic strains have usually ESBL producers that affect hospitalized patients and are leading causes of hospital-acquired infections [5]. *K. pneumoniae* is one of the three major drug-resistant bacteria on the WHO priority list that requires more research and production of novel antibiotics for the treatment [1, 2]. Extensive drug resistance (XDR) against different classes of antibiotics had converted this organism to a big bug in health-care settings, which in turn limits treatment options of infections due to *K. pneumoniae* [1, 4]. This bacterium uses various resistance mechanisms including destructive enzyme production, efflux pumps, porin loss, and target alteration to counteract the effects of antibiotics [1, 3, 6]. So, nosocomial infections with multidrug-resistant (MDR) strains of *K. pneumoniae* are occurring with high mortality and morbidity [3, 4]. *K. pneumoniae* similar to other opportunistic pathogens affect patients who have predisposing debilitating backgrounds and are mainly reported from ICUs, urinary tract infections, ventilator-associated pneumonia, and sepsis [1, 3, 5, 7, 8].

This bacterium has to carry a collection of antibiotic genes with chromosomal and plasmid origins. Acquiring antibiotic resistance genes with plasmid and other transposable agents and mutations after antibiotic pressure has produced superresistant strains of high-risk MDR and XDR *K. pneumoniae* isolates in clinical settings especially in the two past decades which have high distribution potential worldwide [4]. The correct and prompt treatment of infections due to *K. pneumoniae* is vital. The resistance mechanisms epidemiology should be evaluated comprehensively in hospital settings worldwide. Based on the critical literature reviews in the different data sources, the antibiotic resistance profile of *K. pneumoniae* isolates as a worldwide threat in the northwest of Iran (Azerbaijan province) was not clear. Therefore, we conducted this study to consider the molecular epidemiology of drug resistance in clinical isolates of *K. pneumoniae*.

## 2. Materials and Methods

### 2.1. Bacterial Isolates

A total of 100 nonduplicated *K. pneumoniae* isolates were obtained from the different wards of Azerbaijan state hospitals, Iran, from 2019 to 2020. All clinical samples were isolated aseptically from the different organs. Presumptive colonies were further studied by the standard conventional methods [9]. The current study was approved by the research ethics committee (IR.TBZMED.REC.1399.745) at Tabriz University of Medical Sciences, Tabriz, Iran.

### 2.2. Antibiotic Susceptibility Testing

Antibiotic susceptibility testing was prepared by the disk diffusion agar method (DDA) based on the CLSI guidelines. The disks from two groups of beta-lactam and non-beta-lactam drugs were used including cefoxitin (30 *µ*g), ceftazidime (30 *µ*g), cefotaxime (30 *µ*g), ertapenem (10 *µ*g), imipenem (10 *µ*g), meropenem (10 *µ*g), gentamicin (10 *µ*g), amikacin (30 *µ*g), ciprofloxacin (5 *µ*g), ofloxacin (5 *µ*g), gatifloxacin (5 *µ*g), kanamycin (30 *µ*g), tobramycin (10 *µ*g), ampicillin (10 *µ*g), amoxicillin-clavulanate (20/10 *µ*g), tetracycline (30 *µ*g), doxycycline (30 *µ*g), tigecycline (15 *µ*g), trimethoprim-sulfamethoxazole (1.25/23.75 *µ*g), fosfomycin (200 *µ*g), aztreonam (30 *µ*g), levofloxacin (5 *µ*g), moxifloxacin (30 *µ*g), ceftriaxone (30 *µ*g), and piperacillin-tazobactam (100/10 *µ*g) (Mast, UK). The results of the DDA method on Muller–Hinton agar plates were analyzed according to the Clinical and Laboratory Standards Institute (CLSI) guidelines [10].

To determine the MICs, the agar dilution was used for ciprofloxacin, levofloxacin, gentamicin, kanamycin, tobramycin, amikacin, cefotaxime, ampicillin, tetracycline, co-trimoxazole, fosfomycin, and imipenem recommended by CLSI [11]. In the current study, the results of susceptibility testing were confirmed by the *Escherichia coli* ATCC 25922 and *Pseudomonas aeruginosa* ATCC 27853 as positive controls of susceptibility testing.

### 2.3. ESBL by the Phenotypic Tests

CLSI recommended combination disk with ceftazidime (30 *µ*g) and cefotaxime (30 *µ*g) disks alone and in mixture with clavulanate (10 *µ*g). A ≥ 5 mm increase in the zone diameter for cefotaxime or ceftazidime in combination with clavulanate versus the zone diameter of the corresponding antimicrobial agent alone defined an ESBL producer [9].

### 2.4. Determination of Carbapenemases by the Phenotypic Tests

To detect carbapenemases, a phenotypic technique was carried out according to the previous described study [12]. All the carbapenem-resistant isolates were selected for more assessment by two methods, the modified Hodge Test (MHT) and the Carba NP test [13, 14].

### 2.5. DNA Extraction and the PCR for Evaluation of the Resistance Genes

The DNA was taken out by the CTAB method as defined previously [15]. PCR assays were carried out by primers specific for the *bla*_NDM,_*bla*_VIM_, *bla*_IMP_, *bla*_KPC_, and *bla*_OXA-48-like_ carbapenemases-encoding genes and *ompK*35 and *ompK*36 as porin-coding genes. Resistance genes of different classes including ESBLs (*bla*_TEM_, *bla*_SHV_, and *bla*_CTX-M_), carbapenemases (KPC, MBL, or both MBL and KPC, AmpC); fluoroquinolones, tetracycline, sulfonamides/trimethoprim, aminoglycosides, and fosfomycin were detected by the PCR. The PCR products were analyzed by electrophoresis in 1.5% agarose gel, and after staining with 0.5 *µ*g/mL safe stain, they were imagined underneath ultraviolet (UV) light. *Pseudomonas aeruginosa*, *E. coli,* and *K. pneumoniae* were used as controls in the current study. Primers used in the current study selected from previous studies [16–31].

### 2.6. Statistical Analysis

The results were studied by the descriptive statistics in the SPSS software for Windows (version 23 SPSS Inc., Chicago, IL, USA).

## 3. Results

### 3.1. Bacterial Isolates

From 2019 to 2020, a total of 100 nonduplicated *K. pneumoniae* isolates were collected from the different wards of Azerbaijan state hospitals, Iran. The number of bacteria isolated according to each clinical specimen included the wound (23%), blood (14%), urinary tract (49%), and pulmonary (14%). The mean age of patients was 38 ± 21. These patients (47 males and 53 females) were hospitalized in the pediatrics wards (5%), burn (12%), surgery (14%), ICU (25%), and internal (44%).

### 3.2. Antibiotic Susceptibility Patterns

**Figure 1:**
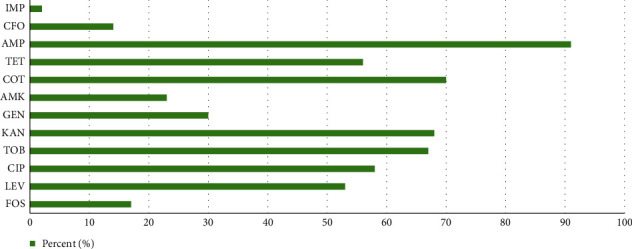
Antibiotic susceptibility testing by the agar dilution. CIP: ciprofloxacin; LEV: levofloxacin; GEN: gentamicin; KAN: kanamycin; TOB: tobramycin; AMK: amikacin; CFO: cefoxitin; IMP: imipenem; AMP: ampicillin; TET: tetracycline; COT: co-trimoxazole; and FOS: fosfomycin.

According to the disk diffusion agar, the frequency of resistance to ciprofloxacin, levofloxacin, ofloxacin, moxifloxacin, gatifloxacin, gentamicin, kanamycin, tobramycin, amikacin, cefotaxime, ceftriaxone, ceftazidime, cefoxitin, ertapenem, meropenem, imipenem, ampicillin, amoxicillin- clavulanate, tetracycline, doxycycline, tigecycline, co-trimoxazole, fosfomycin, aztreonam and, piperacillin-tazobactam was 67%, 39%, 42%, 54%, 47%, 51%, 49%, 51%, 14%, 68%, 67%, 68%, 28%, 33%, 25%, 25%, 96%, 77%, 41%, 54%, 9%, 63%, 11%, 65, and 72%. In the present study, 85% of isolates were resistant to at least three classes of antimicrobial agents considered as an MDR. Of the MDR isolates, sixty-five (65%) were ESBLs-producer. The results of MIC method were consistent with the DDA method except slightly different (Figure 1). Wholly, the carbapenem-resistant isolates were confirmed by two methods, the modified Hodge test and Carba NP test.

### 3.3. Molecular Epidemiology

The fluoroquinolone resistance gene was found in 49 (49%) isolates, and the most common *qnr* gene was *qepA* in 95% (46 of 49), followed by *oqxB* in 87.5% (43 of 49) and *oqxA* in 70% (34 of 49).  Table 1 shows the distribution of *qnr* genes among *K. pneumoniae* isolates. Among the 100 *K. pneumoniae* isolates, 52 (52%) isolates were resistant to tetracycline, as determined via the DDA technique. The PCR detected *tetB* in 42% (22 of 52) isolates, *tetA* in 32% (17 of 52), *tetD* in 21% (11 of 52), and *tetC* in 16% (8 of 52). Seventy isolates (70%) were resistant to sulfonamides/trimethoprim. We discovered *sul*1 in 71% (50 of 70) isolates, *sul*2 in 43% (30 of 70), *dfr* in 29% (20 of 70), and *sul*3 *in* 7% (5 of 70). In the current study, 54 (54%) isolates were resistant to aminoglycosides. The most common resistance gene was *ant*3*Ia in* 44% (24 of 54), followed by *aac*6*Ib* (32%, 17 of 54), *aph*3*Ib* (32%, 17 of 54), and *APHs* (31.4%, 16 of 54). Sixty-five isolates (65%) were phenotypically detected as ESBL. The most frequent ESBL gene was *bla*_SHV-1_ 58% (38 of 65), followed by *bla*_CTX-15_ 55% (36 of 65) and *bla*_SHV-11_ 42% (27 of 65). Sixteen (16%) isolates were resistant to fosfomycin. The most frequent resistance genes in the tested isolated were *fosA* (40%, 6 of 16) and *fosX* (40%, 6 of 16), followed by *fosC* (20%, 4 of 16) (Table 1).

## 4. Discussion

Nosocomial infections have been a major challenge for clinicians worldwide, especially in developing countries. *K. pneumoniae* is one of the most common nosocomial infection agents in hospitals. Increasing resistance to antimicrobial agents has not only increased the duration of the hospitalization but also increases the cost of treatment. Despite a huge number of studies on antimicrobial profiles of resistance and MDR from clinical settings in different provinces of Iran, the comprehensive epidemiology of *K. pneumoniae* resistance genes is not clear yet. Literature review of resistance patterns of *K. pneumoniae* isolates indicates that different rates of resistance and related genes existing in health-care settings in the world. Certainly, this study could help to prepare a consensus on the extensive analysis of resistance gene maps and implement control and treatment programs against such threatening infections in the future.

In the current study, positive ESBLs phenotype was observed in 65%, and the MDR producing *K. pneumoniae* isolates was 85%. The most frequent ESBL gene in the tested isolates was *bla*_SHV-1_ in 58% followed by *bla*_CTXM-15_ in 55% and *bla*_SHV-11_ in 42%. In a study from South Africa, 202 *Klebsiella* spp. were collected; 83.7% were identified as *K. pneumoniae* and 57.9% as other *Klebsiella* spp. The isolates were studied for their resistance patterns and presence of ESBLs and carbapenemases genes. They reported that *bla*_SHV_ (77.1%) was the most highly prevalent ESBL gene, followed by *bla*_TEM_ (66.9%) and *bla*_CTXM_ (56.7%) [32]. Ferreira et al. in a study on the clinical *K. pneumoniae* isolates in the ICU explained that 84% of isolates were MDR with a high rate of resistance against *β*-lactams, quinolones, aminoglycosides, tigecycline, and colistin. They found that all of the isolates were as ESBLs and carbapenemases-producers which harboring *bla*_KPC_ (100%), *bla*_TEM_ (100%), *bla*_SHV_ variants (96%), *bla*_OXA-1_ group (84%), and *bla*_CTXM-1_ group (72%) genes [3]. In the present study, 85% of isolates were resistant to as a minimum of three antibiotics classes considered as an MDR, and ESBLs phenotype was observed in 65 isolates, similar to previous studies [3, 32]. The high frequency of ESBL-producing *K. pneumoniae* may be due to arbitrary consumption of third-generation cephalosporin in the society [33]. According to the results of the antibiotic susceptibility patterns, the highest frequency of resistance was found to ampicillin. This finding is similar to another study from Iran [34] and Russia [5]. Tigecycline and fosfomycin were the most effective antibiotics in this study. Carbapenem has been reflected as an important option for the treatment of resistant Enterobacteriaceae particularly MDR and ESBLs producing isolates. The emergence of carbapenem-resistant Enterobacteriaceae was reported following the increased use of carbapenem [35]. In our study, the resistance to meropenem and imipenem was observed in 25% by the DDA. Other studies also reported carbapenem-resistant Enterobacteriaceae isolates from China and Iran [7, 34, 36]. Xu et al. in a study on 89 *K. pneumoniae* isolated from ventilator-associated pneumonia reported that 33.7% were ESBL producers which were simultaneously MDR. These isolates had genes of *bla*_SHV_, *bla*_CTXM_, *bla*_OXA_, and *bla*_TEM_ with 70%, 70%, 3.3%, and 66.6%_,_ respectively. Interestingly, *bla*_CTXM-15_ was reported as the highest frequent ESBL gene in China [8]. The resistance profile of *K. pneumoniae* isolates from Iranian provinces is relatively different. According to the Kiaei et al. from south of Iran (Kerman) from 175 clinical *K. pneumoniae* isolates, 41.1%, 6.8%, and 21.1% were positive as ESBLs, AmpC, and carbapenemases, respectively; also, 4.5% was both ESBL and AmpC positive. Very interestingly, the resistance rate against carbapenem including imipenem (25.7%) and meropenem (18.9%) was higher than ciprofloxacin at 17.7%. The frequency of ESBL genes was reported as follows *bla*_CTXM_ (46.28%), *bla*_SHV_ (41.1%), *bla*_TEM_ (38.9%), and *bla*_OXA-1_ (21.7%). *bla*_NDM_ was the only carbapenemases gene reported in 21.14% of isolates [6]. The occurrence of *bla*_SHV_ and *bla*_CTXM_ genotypes greatly varies geographically among ESBL-producing isolates. A previous study revealed that *bla*_SHV_ and *bla*_CTXM_ variants were the most common among the ESBLs gene [6, 8, 32]. The results of the present study are also close to the previously mentioned studies.

Tetracycline resistance can be mediated by ribosomal protection, efflux pumps, or chemical modification. In the present study, the resistance rate to tetracycline and doxycycline by the DDA assay was 41% and 54%, respectively. The frequency of tetracycline resistance was reported differently. Tetracycline resistance rate was reported to be 27.5 to 50% among Enterobacteriaceae from Saudi Arabia [37]. In a previous study, the tetracycline resistance among *K. pneumoniae* was reported at 41.3% from Tabriz, Iran [34]. In another study from Tehran hospital, 64% of *K. pneumoniae* were isolated from UTIs carrying *tet* gene (highest antibiotic resistance), and the lowermost resistance (2%) was reported to imipenem and gentamicin [37]. The difference in the frequency of resistance to tetracycline may be due to the geographic regions, amount of antibiotic usage, continued prescribing of tetracycline in veterinary, and a difference in a program of infection control. In the present study, *tetA*, *tetB*, *tetC*, and *tetD* were detected in 32%, 42%, 16%, and 21% of resistance isolates, respectively. Another study from Tabriz, Iran, reported *tetA*, *tetB*, *tetC,* and *tetD* in 14.4%, 18.4%, 2%, and 4.4%, respectively [34]. Both *tetA* and *tetB* genes generally were reported as the most common *tet* genes among tetracycline-resistant Enterobacteriaceae [38–40].

The previous studies indicated that fosfomycin is a broad-spectrum antibiotic against Gram-negative and Gram-positive bacteria [41]. Recently, much attention has been paid to fosfomycin for high clinical value in the treatment of MDR Enterobacteriaceae [42]. However, with the increased application of fosfomycin, resistant isolates have been reported. Multiple mechanisms of fosfomycin resistance can be due to antimicrobial-modifying enzymes, target site modification, or decreased permeability [43]. In the current research, the resistance rate to fosfomycin was 16%. In a previous study from Iran, the resistance rate to fosfomycin in *K. pneumoniae* was 10.9% [44], and these findings are similar to our study. Also, the obtained results of resistance to fosfomycin in both the DDA and agar dilution methods in the current study are in accordance, only the slight and neglectable difference was observed. In the present study, the dominant founded resistance genes to fosfomycin were *fosA*, *fosC*, and *fosX* in 40%, 20%, and 40%, respectively. These results contradict some previous studies in Iran [44], and the percentage differs slightly in the world. The trivial difference in the obtained statistics can be explained according to the number of specimens, the health status of countries, and the pattern of prescribed antibiotics in the various reagents. On the other hand, the identification of *fosX* gene is of the utmost importance in our study because of the scarcity of this gene in the previous investigations. Although fosfomycin has dramatically antibacterial activity, the trivial identified resistance cases are an alarm because of the determinant locations on mobile genetic elements like the plasmids.

Co-trimoxazole is a combination of the antifolate compounds trimethoprim and sulfamethoxazole, which have a synergistic effect, and has been used as a broad-spectrum antibiotic [45]. Co-trimoxazole has been used for several decades as an efficient antibiotic for the treatment of enteric bacterial and urinary tract infections [46]. The injudicious use of co-trimoxazole in developing countries has been a major factor in the emergence of decreased susceptibility to this antibiotic [47, 48]. Three plasmid-mediated dihydropteroate synthase genes encoding sulfonamide resistance, *sul*1, *sul*2, and *sul*3, have been identified in Gram-negative bacteria and more than 30 *dfr* gene encoding resistant variants to trimethoprim have been described [46, 49]. In the current research, co-trimoxazole is the second most common drug-resistant (70%) which was consistent with previous studies in Iran and another country [5, 34]. Based on this study, the *sul*1 gene has the highest frequency in *K. pneumoniae* resistance to co-trimoxazole. The rate of *sul*1 (71%) was higher than *sul*2 (43%) and *sul*3 (7%), which is in accordance with a study from Tanzania [50]. The frequency of *sul* genes in a previous study in Iran on *Escherichia coli* isolates was close to our study [51]. The existence of *sul* genes in different environmental and clinical isolates shows that these genes have a general role in carrying and distributing sulfonamide resistance in bacteria [52–54]. The *dfr* gene in our research was detected in 29% of *K. pneumoniae* isolates. The high rate of *sul* genes and high occurrence of co-trimaxazole resistance in *K. pneumoniae* isolates points toward that uninterrupted investigation programs should be applied in clinical settings to better control and manage associated diseases and monitor the trends of co-trimaxazole resistance in Gram-negative bacteria.

Fluoroquinolone is the most significant antibiotic used for the management of bacterial infections [55]. Newly, fluoroquinolone resistance has increased in clinical isolates. The most common fluoroquinolone resistance mechanism is due to the chromosomal mutations in quinolone resistance determining regions (QRDR) and plasmid-mediated quinolone resistance (PMQR) [56]. In the present study, 49 isolates contain fluoroquinolone resistance genes. As formerly revealed, PMQR genes play a significant role in resistance to quinolone due to parallel transferability [57]. In our study, the most prevalent PMQR gene was *qepA* (95%) followed by *oqxB* (87.5%), *oqxA* (70%), and *qnrB* (27.5%). Contrary to our study, another study in Iran on *K. pneumoniae* reported that the most predominant PMQR gene was *oqxA* followed by *oqxB* and *qepA* [58]. In this study, *qnrB* gene (27.5%) was more prevalent among *qnr* genes and the lowest rate was *qnrA* (5%). Interestingly, in some studies, *qnrA* was not found. Our results are constant with the results of previous studies [59–61]. In our study like some research, all three resistant genes (*qnrB*, *qnrA*, and *qnrS*) have also been originated in clinical isolates [60, 62]. The types of *qnr* genes may vary in different geographical locations [63]. One of the important features of this research is the observation of *qnrD* (25%) in clinical isolates. The present study established a high prevalence of *qepA* (95%), *oqxB* (87.5%), and *oqxA* (70%) genes among PMQR factors. The transferability amount of these factors is high. This is a reason for worry since the horizontal transfer of PMQR genes can raise the spread of resistance to fluoroquinolone.

Aminoglycosides are one the most essential treatments used for *K. pneumoniae* infections. The mechanisms of resistance to aminoglycosides include modification of the ribosomal target, enzymatic modification, and diminished intracellular antibiotic accumulation by changes of the outer membrane permeability, reduced inner membrane transport, or active efflux pumps. Adenylation and acetylation are two main mechanisms of aminoglycoside resistance, catalyzed by actyl-CoA in need of N-acetyltransferase and ATP-dependent O-nucleotidyltransferase, respectively [64, 65]. In the current study, a total of 54 aminoglycoside-resistant *K. pneumoniae* strains were found among 100 clinical isolates. Our results showed that the resistance of *K. pneumoniae* isolates to gentamicin, kanamycin, tobramycin, and amikacin was 51%, 49%, 51%, and 14%, respectively. Our results showed that the rate of resistance to gentamicin, tobramycin, and amikacin was consistent with another research from Iran [66]. Interestingly, the results of Hesam et al. study about the rate of resistance to gentamicin and tobramycin were very close to our study [66]. In our research, according to the DDA method, the highest aminoglycosides resistance among *K. pneumoniae* isolates was related to gentamicin and the lowest resistance was related to amikacin. These results were consistent with El-Badawy et al., but the rate of resistance to amikacin was approximately doubled in their study [61]. The findings of this study showed that the most frequent aminoglycosides resistant genes were *ant* (3) *Ia* (44%) followed by *aac* (6) *Ib* (32%), *aph* (3) *Ib* (32%), and *APHs* (31.4%). In another study on *Klebsiella* spp. and *E. coli* isolated from Egypt, the most dominant aminoglycoside-modifying enzymes (AMEs) were *aac* (3) *IIa* and *aac* (6) *Ib*. On the other hand, their research specified that none of *aac* (3) *Ia* and *rmt55* genes were distinguished in any of *Klebsiella* spp. and *E. coli* isolated from Egyptian patients [67]. Our results were consistent with a study in Egypt, but contrary to their research, the highest presence of AME genes in the current study was related to *ant* (3) *Ia*. In the study of Iraj et al., the *aac* (3) *IIa*, *aac* (6) *Ib,* and *rmtB* genes were identified in 55.8%, 80.8%, and 1.9% of the isolates, respectively. The *aac* (3) *Ia* and *armA* were not found in any of the isolates [68]. In the previous studies from Spain by Elisenda et al., the most frequent AME genes in Enterobacteriaceae were *aph* (3) *Ib* (65.4%) and *ant* (3) *Ia* (37.5%) [69], whereas in the recent study, the most frequent AME genes in *K. pneumoniae* were *ant* (3) *Ia* (44%) followed by *aac* (6) *Ib* (32%) and *aph* (3) *Ib* (32%). This could be due to the diversity of bacterial species used in the Spanish study. The rate of AAC (3)-II has diminished. In 1993, Shaw et al. defined an occurrence of AAC (3)-II in 60.3% of 2445 Gram-negative bacteria [70]. However, we found a prevalence of AAC (3)-II in (24%). In a previous study, the most predominant enzyme was AAC (6)-Ib [70]. However, these enzymes are essential for the reason that they are the few genes that confer resistance to amikacin and quinolones. The results of our study also confirmed the issue. Our results showed high frequencies of aminoglycosides resistance genes in *K. pneumoniae* isolates which could be the results of unnecessary use of antibiotics, and these outcomes discovered the status of the transfer of antibiotic resistance genes.

In this study, the patterns of antibiotic resistance and the frequency of resistance coding genes in *K. pneumoniae* isolated from Azerbaijan state hospitals by phenotypic and molecular methods were studied. So, the determination of the patterns of antibiotic resistance in common pathogenic bacteria including *K. pneumoniae* is important to guide empirical therapy against a particular pathogen; also, it helps reduce treatment costs and length of hospital stay; moreover, it may be used as a warning for officials and policymakers in the national and provincial health basin to take more seriously the issue of the emergence incidence of drug resistance among bacteria especially *K. pneumoniae*. The routine usage of antibiotics in the food business, especially in the agricultural and livestock industry, is recommended monitor by health officials. Furthermore, choosing an antibiotic by antibiotic susceptibility testing and continuous monitoring of drug resistance could have a key role in the management of infectious diseases and reduce antibiotic resistance. Due to the high occurrence of drug resistance in Azerbaijan hospitals, it is recommended to conduct a national study in Iran, to evaluate the rate of antimicrobial resistance in *K. pneumoniae*.

## 5. Conclusion

Antimicrobial resistance in *K. pneumoniae* has become one of the most challenging issues faced in daily clinical practice. Our results indicated a high occurrence of drug resistance among *K. pneumoniae* isolates in Azerbaijan hospitals. The resistance to co-trimoxazole is the most common, followed by aminoglycosides, tetracycline, fluoroquinolone, and fosfomycin. This is due to the presence of high levels of drug-resistant genes in various antibiotics, which are frequently used in the treatment of infections caused by *K. pneumoniae.*

## Figures and Tables

**Table 1:** Distribution of antibiotic resistance genes in the current study.

Antibiotics	Genes conferring resistance (%)
*Fluoroquinolone* Ciprofloxacin, ofloxacin, gatifloxacin, levofloxacin, and moxifloxacin	*qepA* (95%), *oqxB* (87.5%), *oqxA* (70%), *qnrA* (5%), *qnrB* (27.5%), *qnrD* (25%), and *qnrS* (17.5%)

*Tetracycline* Tetracycline, doxycycline, and tigecycline	*tetA* (32%), *tetB* (42%), *tetC* (16%), and *tetD* (21%)

*Co-trimoxazole*	*dfr* (29%), *Sul*3 (7%), *Sul*2 (43%), and *Sul*1 (71%)

*Aminoglycosides * Gentamicin, amikacin, kanamycin, and tobramycin	*APHs* (31.4%), *ANTs* (30.7%), *rmtB* (20%), *aac*3*Ib* (10%), *aac*3*IIa* (24%), *aac*6*Ib* (32%), *ant*3*Ia* (44%), *ant*4*IIa* (5%), *aph*3*Ia* (10%), and *aph*3*Ib* (32%)

*Beta-lactamases* Cefoxitin, ceftazidime, cefotaxime, ertapenem, imipenem, meropenem, ampicillin, ceftriaxone, amoxicillin-clavulanate, piperacillin-tazobactam, and aztreonam	*bla*_TEM-1_ (40%), *bla*_TEM-16_ (33%), *bla*_TEM-24_ (3%), *bla*_SHV-1_ (58%), *bla*_SHV-11_ (42%), *bla*_CTXM-15_ (55%), *bla*_CTXM-3_ (27%), *bla*_CTXM-1_ (5%), *bla*_CTXM-79_ (9%), *bla*_CTXM-27_ (5%), *bla*_MBL_ (16%), *bla*_KPC_ (7%), *bla*_OXA-48_ (4%), porin loss (35%), *omp*35 (80%), *omp*36 (77%), and *ampC* (12%)

*Fosfomycin*	*fosA* (40%), *fosX* (40%), and *fosC* (20%)

## Data Availability

The data used to support the findings of this study are available from the corresponding author upon request.
